# An efficient method for high molecular weight bacterial DNA extraction suitable for shotgun metagenomics from skin swabs

**DOI:** 10.1099/mgen.0.001058

**Published:** 2023-07-10

**Authors:** Iliana R. Serghiou, Dave Baker, Rhiannon Evans, Matthew J. Dalby, Raymond Kiu, Eleftheria Trampari, Sarah Phillips, Rachel Watt, Thomas Atkinson, Barry Murphy, Lindsay J. Hall, Mark A. Webber

**Affiliations:** ^1^​ Quadram Institute Bioscience, Norwich Research Park, Norwich, Norfolk, NR4 7UQ, UK; ^2^​ School of Biological Sciences, University of East Anglia, Norwich Research Park, Norwich, Norfolk, NR4 7TJ, UK; ^3^​ Unilever R&D Port Sunlight, Bebington, CH63 3JW, UK; ^4^​ Norwich Medical School, University of East Anglia, Norwich Research Park, Norwich, Norfolk, NR4 7TJ, UK

**Keywords:** Long and short read sequencing, Microbial abundance, Skin microbiome

## Abstract

The human skin microbiome represents a variety of complex microbial ecosystems that play a key role in host health. Molecular methods to study these communities have been developed but have been largely limited to low-throughput quantification and short amplicon-based sequencing, providing limited functional information about the communities present. Shotgun metagenomic sequencing has emerged as a preferred method for microbiome studies as it provides more comprehensive information about the species/strains present in a niche and the genes they encode. However, the relatively low bacterial biomass of skin, in comparison to other areas such as the gut microbiome, makes obtaining sufficient DNA for shotgun metagenomic sequencing challenging. Here we describe an optimised high-throughput method for extraction of high molecular weight DNA suitable for shotgun metagenomic sequencing. We validated the performance of the extraction method, and analysis pipeline on skin swabs collected from both adults and babies. The pipeline effectively characterised the bacterial skin microbiota with a cost and throughput suitable for larger longitudinal sets of samples. Application of this method will allow greater insights into community compositions and functional capabilities of the skin microbiome.

## Data Summary

All sequence data and codes can be accessed at:

NCBI Bio Project ID: PRJNA937622

DOI: https://github.com/quadram-institute-bioscience/coronahit_guppy

DOI: https://github.com/ilianaserghiou/Serghiou-et-al.-2023-Codes

**Impact Statement**

Determining the functional capabilities of microbial communities within different human microbiomes is important, to understand their impacts on health. Extraction of sufficient DNA is challenging, especially from low biomass samples, such as skin swabs, suitable for shotgun metagenomics, which is needed for taxonomic resolution and functional information. Here we describe an optimised DNA extraction method that produces enough DNA from skin swabs, suitable for shotgun metagenomics, and demonstrate it can be used to effectively characterise the skin microbiota. This method will allow future studies to identify taxonomic and functional changes in the skin microbiota, which is needed to develop interventions to improve and maintain skin health.

## Introduction

The skin microbiome is a complex ecosystem organised into distinct microbial communities present at different body sites [1, 2]. These microbial ecosystems participate in the host’s skin physiological functions and immunity [3, 4]. Perturbations in these communities can negatively impact skin health, particularly early in life [5]. Studying how the skin microbiota forms and changes over time is therefore important to understand how interventions that alter the microbiota affect skin health.

Previous skin microbiome studies have commonly used traditional 16S rRNA gene amplicon sequencing (metataxonomics) to taxonomically classify these complex communities [6]. Whilst relatively cheap, 16S rRNA gene amplicon sequencing provides limited taxonomic information on bacteria and archaea, and some variations in this method can identify species level, but cannot provide information about strain variations or functional capacities. Alternatively, the use of Shotgun Metagenomic Sequencing (SMS) for taxonomic classification follows sequencing of all genetic material and is not limited to targeted regions [7–9]. This reduces bias from selective amplification efficiency and can provide taxonomic information at species/strain level as well as being able to provide information about functional capacities present in the microbiome and individual species [6, 9, 10]. SMS can be performed using multiple technologies, including the Illumina, Oxford Nanopore (ONT) and PacBio Single Molecule Real-Time (SMRT) platforms [11, 12]. In contrast to the Illumina technology, the ONT and PacBio SMRT technologies produce long sequence reads. Data produced with these platforms will usually reconstruct more complete genomes than from short reads and facilitates the generation of high-quality Metagenome Assembled Genomes (MAGs) [12], which can be used for higher taxonomic resolution and functional information [10, 13].

The relatively low bacterial biomass of skin complicates the extraction of sufficient DNA quantities for SMS [14, 15]. This is particularly true for longer read technologies where more input material is needed [16]. There are a limited number of commercialised kit protocols available that can produce high molecular weight (HMW) DNA from skin in sufficient quantities for SMS, although none have been specifically optimised within a protocol that includes sample collection, lysis, DNA extraction, then informatic analysis from skin microbiome samples. To address this need we describe here an optimised high-throughput automated DNA extraction method, for recovery of HMW microbial DNA from skin swabs. This was validated using skin swabs from adult volunteers and babies enrolled in the Pregnancy and Early Life (PEARL) study [17]. The method results in DNA with yield and molecular weight suitable for SMS.

## Methods

### DNA extraction method development

To optimise extraction of microbial DNA from skin swabs, different diluents and lysis procedures were evaluated for effectiveness before DNA was extracted using a Promega Maxwell RSC 48 Instrument and RSC Blood DNA Kit (see File S1, available in the online version of this article for protocol). This instrument and kit were chosen as they produce HMW DNA [18, 19], with a higher binding capacity and cleaner eluate than traditional silica-based DNA purification systems [20–23]. The platform is also suitable for high-throughput automated genomic DNA isolation capable of processing from 48 samples in 40 min [22] making this system compatible with larger sample sets. The focus of this work was to develop an efficient, optimised, and validated lysis method for recovering DNA from skin microbiota. Although we developed this using the Maxwell platform, the method should be applicable to any other automated and column-based kits on the market, our goal was not however to benchmark extraction kits, which was beyond the scope of producing this protocol.

**Fig. 1.**
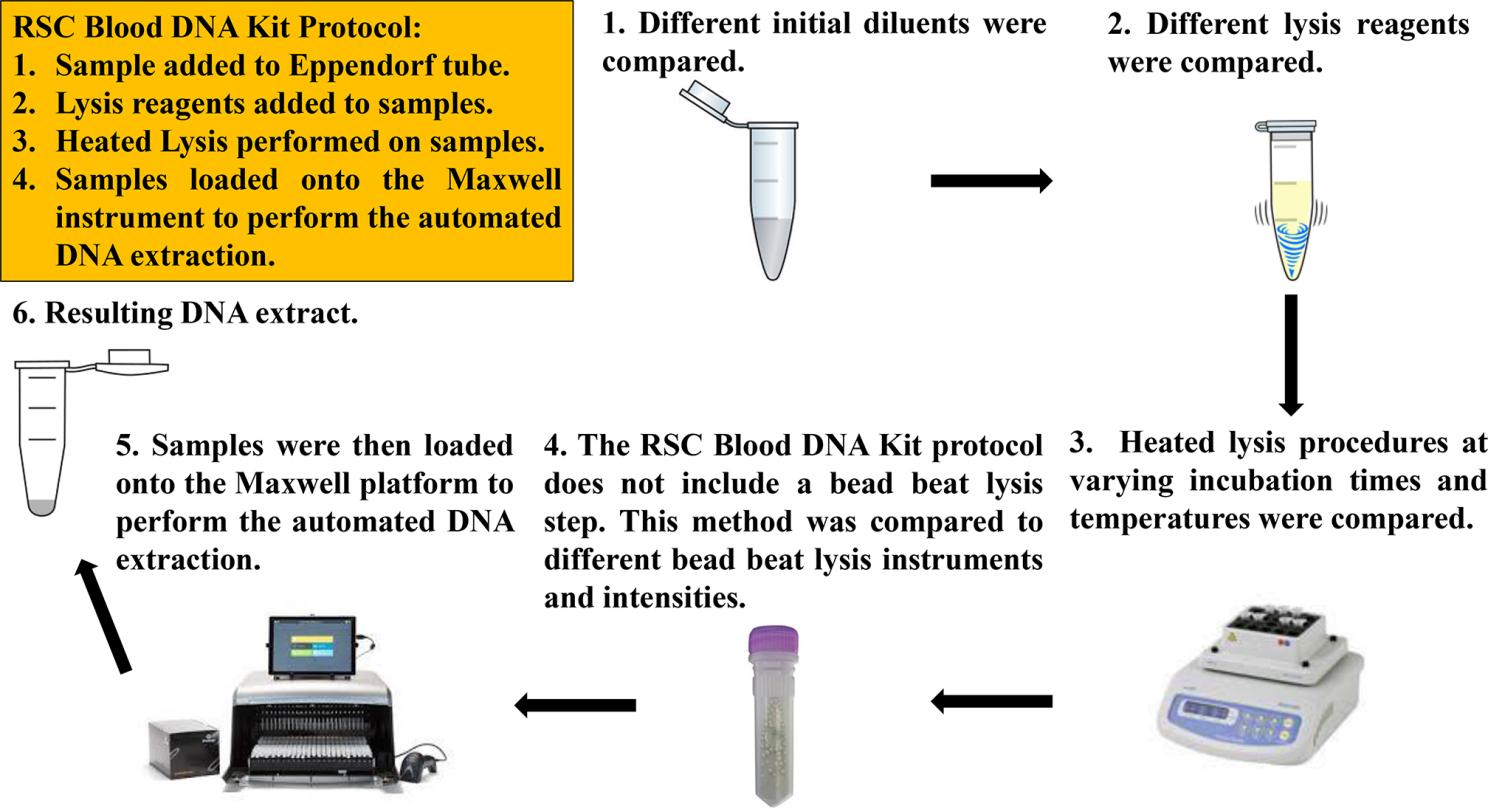
The RSC Blood DNA Kit protocol (yellow box) and alterations to test different initial diluents and lysis procedures.

To obtain enough DNA from skin swabs, suitable for SMS, we optimised the RSC protocol by testing different variables including the initial diluent and various lysis procedures. After dilution and lysis, samples were heated, following the RSC Blood DNA Kit protocol, and loaded to the Maxwell instrument for the automated extraction (Fig. 1).

### Testing initial diluents: measuring extracted bacterial DNA quantity and cellular viability

To allow for a protocol where a swab could be processed allowing both DNA extraction and, in parallel, culture of organisms, it was desirable to remove material from the swab into a diluent. To determine if diluents impacted bacterial viability and ability to extract DNA, 1 x Phosphate Buffered Saline (PBS) and Ultrapure Milli-Q water, for collecting skin bacteria, were compared by measuring extracted bacterial DNA quantity recovered from swabs inoculated with bacteria. Fourty-four sterile charcoal cotton swabs (M40-A2, Technical Service Consultants Ltd.) were used to collect a single colony from an agar plate inoculated with *Staphylococcus aureus* NCTC 8532 to act as a target for DNA extraction. These ‘spiked’ swab heads were snapped into 1.5 ml Eppendorf tubes containing 1 ml of either 1 x PBS or Ultrapure Milli-Q water. These were then extracted following the Promega Maxwell RSC 48 Instrument and RSC Blood DNA Kit protocol in File S1, with the following modification: the swabs were vortexed at full speed for 2 min and then centrifuged at 14000 ***g*** for 15 min to pellet the cells before the supernatant was removed, and cells were resuspended in 300 µl of 1 x PBS or Ultrapure Milli-Q water. Steps 4 and 6–8 of the RSC protocol were then followed. A bead beating step was then performed using the MP Biomedicals FastPrep instrument for 3 min at speed setting 6.0. The samples were centrifuged again at 14000 ***g*** for 15 min to pellet the cells before sample supernatants were loaded onto the Maxwell instrument and the extraction started following steps 9–21 of the RSC protocol.

The effectiveness of 1 x PBS and Ultrapure Milli-Q water, as initial diluents for collecting skin bacteria, were further compared by measuring bacterial cell viability through the recovery of bacteria from liquid cultures. Cell viability is an important factor as we wanted an initial dilution step, which maintained bacterial viability and was therefore compatible with both culture of bacteria from samples and efficient DNA extraction. Overnight liquid cultures (10 ml) were grown from isolates of three species (* S. aureus* NCTC 8532, *Pseudomonas aeruginosa* PA14 and *Escherichia coli* EC18PR-0166–1, a food isolate of ST10), with three replicates for each. For each replicate, 1 ml was transferred into a 15 ml falcon tube and pelleted by centrifugation at 14000 ***g*** for 15 min. Samples were then resuspended in 200 µl of LB, 1 x PBS or Ultrapure Milli-Q water and left for 1 h at ambient temperature. Serial dilutions of the resuspended samples were made and plated onto drug-free agar and incubated, which were then used to count viable numbers of cells in each sample. A total of nine independent samples were tested for each species in each diluent.

### Testing lysis methods: six extraction method procedures

Six lysis methods were compared to identify the best method for high yields of high molecular weight DNA from both Gram-negative and Gram-positive bacteria. Each method varied factors from common lysis methods used in commercial kits for research – heat, chemical, enzymatic, and mechanical [24, 25]. Table 1 lists the differences between the six methods. Methods were tested using both overnight liquid cultures and sterile swab heads inoculated with harvested bacteria from overnight plate cultures.

**Table 1.** Comparison of extraction methods

	*Method 1*	*Method 2*	*Method 3*	*Method 4*	*Method 5*	*Method 6*
***Heated lysis Step***	Yes	Yes	Yes	Yes	Yes	Yes
*Time*	20 mins	20 mins	18 h	18 h	18 h	18 h
*Temperature*	56 °C	56 °C	37 °C	37 °C	37 °C	37 °C
*Reagents*	Proteinase K, lysis buffer	Proteinase K, lysis buffer	Epicentre ready-lyse lysozyme	Epicentre ready-lyse lysozyme	Thermo Fischer lysozyme	Thermo Fischer lysozyme
***Agitation***	No	No	300 r.p.m.	300 r.p.m.	300 r.p.m.	300 r.p.m.
*Bead beat step*	Yes	Yes	Yes	Yes	Yes	Yes
*Instrument*	FastPrep	Tissue Lyser	FastPrep	Tissue Lyser	FastPrep	Tissue Lyser
*Settings*	3 mins at 6.0 FastPrep	3 mins at 20 Hz	3 mins at 6.0 FastPrep	3 mins at 20 Hz	3 mins at 6.0 FastPrep	3 mins at 20 Hz
***Heated offboard lysis step***	No	No	Yes	Yes	Yes	Yes
*Temperature*	n/a	n/a	68 °C	68 °C	68 °C	68 °C
*Time*	n/a	n/a	15 mins	15 mins	15 mins	15 mins
*Reagents*	n/a	n/a	Proteinase K, buffer ATL, carrier RNA, buffer ACL	Proteinase K, buffer ATL, carrier RNA, buffer ACL	Proteinase K, buffer ATL, carrier RNA, buffer ACL	Proteinase K, buffer ATL, carrier RNA, buffer ACL
*Agitation*	n/a	n/a	300 r.p.m.	300 r.p.m.	300 r.p.m.	300 r.p.m.

Duplicate 10 ml overnight liquid cultures were grown for each species (* S. aureus *, *P. aeruginosa* and *E. coli *), from each, 300 µl was added into two 1.5 ml Eppendorf tubes resulting in six tubes, which were tested for method 1 and 2. A further 400 µl of each liquid culture was added into four tubes resulting in six tubes tested for each remaining method. All samples were then extracted following the Promega Maxwell RSC 48 Instrument and RSC Blood DNA Kit protocol (detailed in File S1) with changes to the lysis procedure for each of the six methods tested. All Eppendorf tubes were then vortexed at full speed for 2 min and centrifuged at 14000 ***g*** for 15 min to pellet the cells; the supernatants were removed, and pellets resuspended in 300 µl (methods 1 or 2) or 400 µl (methods 3–6) of 1 x PBS.

For method 1 and 2 samples, 30 µl of Proteinase K and 300 µl of Lysis Buffer (both reagents included in the RSC Blood DNA Kit) were added to the 300 µl sample suspensions. These were then incubated in a heating block at 56 °C for 20 min. For methods 3 and 4 samples, 3 µl of Epicentre Ready-Lyse lysozyme (diluted to 250 U µl^−1^ in Tris-EDTA Buffer [Sigma-Aldrich]) was added to the 400 µl sample suspensions. For methods 5 and 6 samples, 3 µl of Thermo Fischer lysozyme (diluted to 250 U µl^−1^ in Tris-EDTA Buffer [Sigma-Aldrich]) was added to the 400 µl sample suspensions. Samples from methods 3–6 were then incubated with agitation at 300 r.p.m., 37 °C for 18 h. A bead beating step was performed on all samples. Method 1, 3 and 5 samples used the MP Biomedicals FastPrep instrument for 3 min at speed setting 6.0 and method 2, 4 and 6 samples used the Qiagen Tissue Lyser instrument for 3 min at 20 Hz to compare the impact of a less intense bead beating step. An off-board lysis was performed on method 3–6 samples, which included addition of 40 µl proteinase K, 165 µl Buffer ATL, 120 µl Carrier RNA (lyophilised Carrier RNA was resuscitated with Buffer AVE to make a 1 µg µl^−1^ solution), and 315 µl Buffer ACL (all off-board lysis reagents from Qiagen) into the 400 µl sample suspensions. These samples were then incubated at 68 °C for 15 min. Samples from all methods were centrifuged at 14000 ***g*** for 15 min to pellet cells and the supernatants were loaded onto the Maxwell instrument and the extraction started following steps 9–21 of the initial RSC protocol.

After evaluation of the performance of the different methods from cultured cells, method six performed the best (see Results) and was chosen for validation using swab samples. For validation, sterile charcoal cotton swabs (M40-A2, Technical Service Consultants Ltd.) were spiked with one colony from overnight plate cultures of each of the three species and eight independent swabs were processed per species. Swab heads were snapped off into 1.5 ml Eppendorf tubes containing 1 ml of 1 x PBS and samples were vortexed for 2 min before being centrifuged at 14000 ***g*** for 15 min to pellet the cells. The supernatants were removed, and the pellets were resuspended with 400 µl 1 x PBS. The method six procedure was then followed as described above.

### Validation of DNA extraction method using volunteer and PEARL study skin swabs

The optimised DNA extraction method was tested on skin swabs from adults and babies to validate the selected method ability to obtain appropriate bacterial DNA for SMS and confirm data was suitable for analysing the taxonomic profiles of bacterial communities present on skin. Samples were cultured in parallel to DNA sequencing; this allowed us to identify organisms which should be represented in the SMS data whilst also enabling the creation of a skin microbiota culture collection for future functional work with strains of interest. Swabs were cultured aerobically and anaerobically on Columbia blood agar plates as in previous studies [26]. For each swab, cells grown on the aerobic and anaerobic plates were harvested into one glycerol stock, a sample of which was then used for DNA extraction and SMS to compare to results direct from swabs.

### Study design for adult volunteer and PEARL study baby skin swab collection

The Norwich Research Park Biorepository recruited and consented 12 adult volunteers between the age of 23–65. There was no contact between the researcher and participants to ensure anonymity. Eligible volunteer participants had no current skin conditions or had been prescribed antibiotics over the last 3 months. The volunteer participants were provided with Participant Information Sheets (PIS) and were consented with Consent Forms (CF) and provided samples using a self-swabbing protocol under observation and following instruction from Biorepository staff (File S2). The volunteers collected two swabs (using charcoal cotton swabs M40-A2, Technical Service Consultants Ltd.), one from the right arm and one from the left arm, to produce 24 samples in total. Samples were stored in a 4 °C fridge and anonymised with a unique barcode before being collected and tested on the same day swabbing was performed. In addition to the adult volunteers, swabs (using charcoal cotton swabs M40-A2, Technical Service Consultants Ltd.) from the skin of ten babies collected at 4 months of age, as part of the PEARL study, were also included (see Phillips *et al*., [17] for study design and inclusion criteria, and Table S1 for baby participant metadata); the PEARL Study selected the use of charcoal cotton swabs due to their suitability for sampling multiple body sites (i.e. vagina, gut and skin) and long-term preservation of microbes in the −80 degrees Celsius freezer. Charcoal cotton swabs were therefore selected for use in all optimisation steps in this study to avoid a bias from the swabs used within our study and the PEARL study.

### Volunteer and baby skin swab processing and finalised DNA extraction procedure

The skin swabs were processed as described above with the optimised method, a cell-free, diluent-only sample was included as a negative control on each extraction run and an established commercial mock community (the ATCC skin microbiome whole cell mix) was included as a positive control [27]. Dilutions of the positive control microbiome mix were also prepared to validate extraction efficiency and identify a cut-off point of starting material needed for SMS. For full details on the sample processing, DNA extraction protocol and the ATCC positive control protocol, see File S3.

### DNA quantification and quality assessment

A High Sensitivity (HS) assay using the Qubit 2.0 fluorometer instrument and HS Qubit Invitrogen kit, was used to quantify all samples. If a concentration was out of range, i.e. too high, the Broad Range (BR) Qubit assay was used instead, using the Qubit 2.0 fluorometer instrument and BR Qubit Invitrogen kit. Tapestation assays were used to determine DNA molecular weight. A D5000 or HS D5000 Tapesation assay were used with an Agilent 2200 instrument and Agilent D5000 or HS D5000 kits.

### Shotgun metagenomic sequencing using Illumina and Oxford Nanopore Technology

Preparation of libraries for SMS for both Illumina (Illumina DNA Prep Kit: 20018704) and ONT (Illumina DNA Prep: 20 018 704, Tagmentation: 20060059) platforms included DNA normalisation, tagmentation, PCR barcoding, quantification, pooling, and quality control. Samples were then loaded onto the Illumina NextSeq500 Instrument using a Mid-output 300 cycle kit (Illumina Catalogue FC-404–2003) or the MinION flow cell ONT instrument (R9.4.1). The QIB Bioinformatics team converted the Illumina raw data to eight FASTQ files for each sample, and the ONT raw data was converted into FASTQ files using the customised guppy method. All FASTQ files were then run through Fastp (V.0.19.5+galaxy1) [28], which is a quality-filtering tool for FASTQ files that removes adaptors. For full details on the SMS protocol for Illumina and ONT, view File S4.

### Generating taxonomic profiles

All SMS data was automatically deposited in a local instance of IRIDA (irida-19.09.2) [29] and uploaded to the QIB Galaxy platform (V.19.05) [30]. Here, data was cleaned by removing adaptors and trimming reads, and filtered for quality using Fastp (V.0.20.0) (-q 20) [28], before reads mapping against a human reference database (human_20200311) were removed using Kraken2 (V.2.1.1+galaxy0) [31]. Remaining reads were then analysed to obtain microbiota taxonomic profiles using Kraken2 (V.2.1.1+galaxy0) [31] and Bracken (V.2.2) [32].

### Metagenome-assembled genomes (MAG) extraction

Using the trimmed and filtered reads, host-associated (human) sequences were removed via Kneaddata (V.0.10.0) (The Huttenhower Lab [33]) with human genome database (GRCh38.p13) to generate clean fastq reads. Shotgun metagenome raw reads were co-assembled with MEGAHIT (V.1.2.9) [34] prior to extraction of MAGs. The MetaWRAP (V.1.3.2) pipeline [35] was used to extract MAGs based upon metagenome assemblies generated and metagenome clean reads via binning software ‘metaBAT’ (V.2.12.1) [36], ‘MAXBIN2’ (V.2.2.6) [37] and ‘CONCOCT’ (V.1.1.0) [38] using the sub-module ‘binning’. MAGs were then refined using sub-module ‘bin_refinement’ to select the high-quality bins from each sample with completeness >80 % and contamination <10 % using CheckM (V.1.1.3) [39]. All MAGs were taxonomically ranked using gtdb-tk (V.1.5.1) [40] via module gtdbtk classify_wf.

### Data visualisation

R (V.4.1.2) [41] and the package ggplot2 [42] were used to plot taxonomic profiles and alluvial and box plots. GraphPad Prism (V.5.04) [43] was used to generate scatter plots.

### Statistical analysis

Statistical analysis was performed using unpaired t-tests in GraphPad Prism (V.5.04) [43]. A significance level of 0.05 was used to identify results likely to be different.

## Results

### Optimisation of DNA extraction method

#### Impact of initial diluents on extracted bacterial DNA quantity and cell viability

**Fig. 2.**
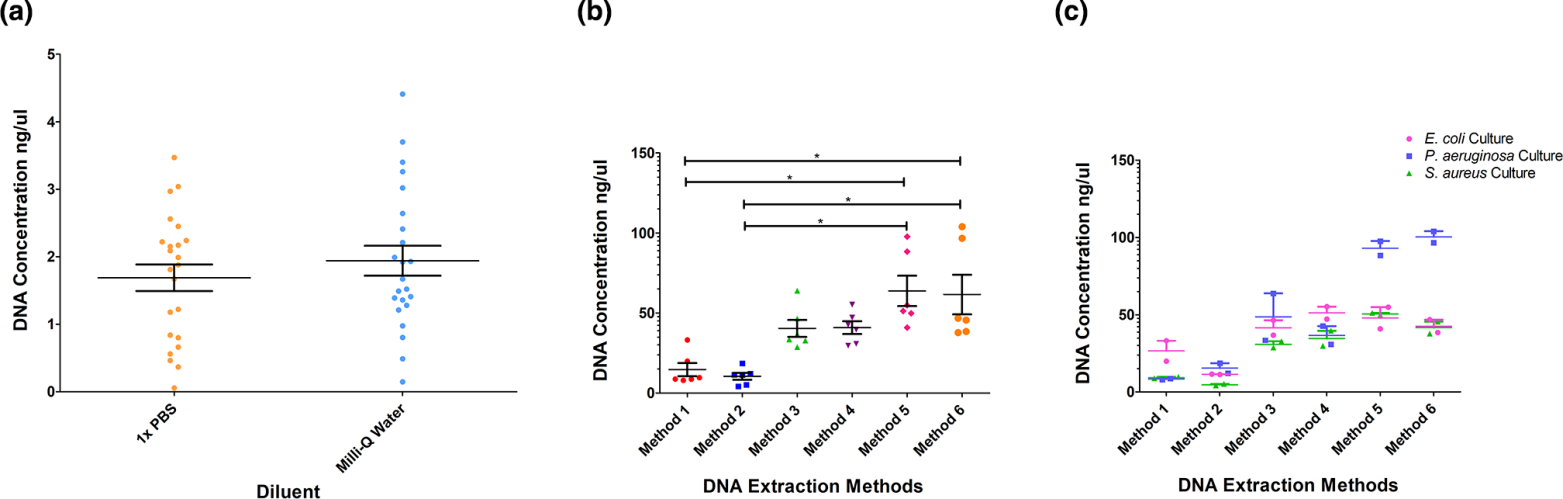
Results of the variables tested. (a): Total DNA yield (ng µl^−1^) from spiked swabs (*n*=44; 22 per diluent) processed in 1xPBS and Milli-Q water during a DNA extraction. (b): Total DNA yield (ng µl^−1^) obtained from each DNA extraction method (*n*=36; six cultures per method). (c): Total DNA yield (ng µl^−1^) per species for each method (*n*=36; six cultures per method). Horizontal bars on each plot show averages, vertical bars show the standard error of the mean (SEM) and lines with an asterisk (*) indicate significant (*P*<0.05) differences.

There was no significant difference between amounts of bacterial DNA extracted from the 44 sterile charcoal cotton swabs spiked with *S. aureus* and processed in either PBS or water (Fig. 2a). Recovery of *S. aureus *, *P aeruginosa* and *E. coli,* also showed no significant differences in viable numbers recovered after suspension in either diluent (*P*>0.05; Fig. S1). As there was no significant difference in both DNA extraction and bacterial recovery between PBS and water, future experiments used PBS.

#### Testing lysis methods: six extraction method procedures

DNA extracted from liquid cultures of *S. aureus *, *P. aeruginosa* and *E. coli* using the six methods (Table 1), showed that methods 5 and 6 yielded the most DNA, (40.9–97.7 ng µl^−1^ and 37–104 ng µl^−1^ respectively), and there was a significant difference in DNA concentrations between methods 5 and 6 and methods 1 and 2 (Table S2; Fig. 2b, c). Methods 5 and 6 yielded higher DNA concentrations for *P. aeruginosa* (Fig. 2c) compared to other species, but there was no statistically significant difference in extraction efficiency between each bacterial species. DNA extraction methods 2, 4 and 6 produced higher molecular weights than the others, ranging from 20232 to 31786 bp (Table 2). Together, these results demonstrated that method six produced the most DNA of highest molecular weight. This method was also the most cost effective due to the cheaper lysozyme used and was chosen for further validation. This method included overnight lysis with lysozyme, a further heated offboard lysis step and a bead beating lysis using the Qiagen Tissue Lyser.

**Table 2.** Average molecular weight (bp) of DNA extracted

Sample	Method 1	Method 2	Method 3	Method 4	Method 5	Method 6
* E. coli *	0	25 321	1735	21 693	1909	24 219
* E. coli *	0	25 697	2057	20 232	1877	22 730
* P. aeruginosa *	0	23 771	1763	23 163	1678	29 459
* P. aeruginosa *	0	23 826	1631	23 742	1538	31 786
* S. aureus *	0	25 085	1831	23 954	1668	22 118
* S. aureus *	0	22 011	1762	24 084	1711	22 904

DNA extractions from sterile charcoal cotton swabs spiked with independent cultures were successful, with DNA concentrations averaged at 22.1 ng µl^−1^.

#### DNA extraction method validation using swabs from volunteers or babies

DNA concentrations from adult and baby skin swabs, that were extracted using method 6, ranged from <0.50 (no detected DNA) – 10.5 ng µl^−1^ (Table S3) with DNA successfully extracted from all the baby samples, but only 15/24 adult volunteer samples. Cultured plates recovered bacteria from all adult skin swabs, although recovery of cultures from the baby samples was only successful for 4/10 swabs. Concentrations of DNA extracted from cultured bacteria averaged at 79.9 ng µl^−1^.

**Fig. 3.**
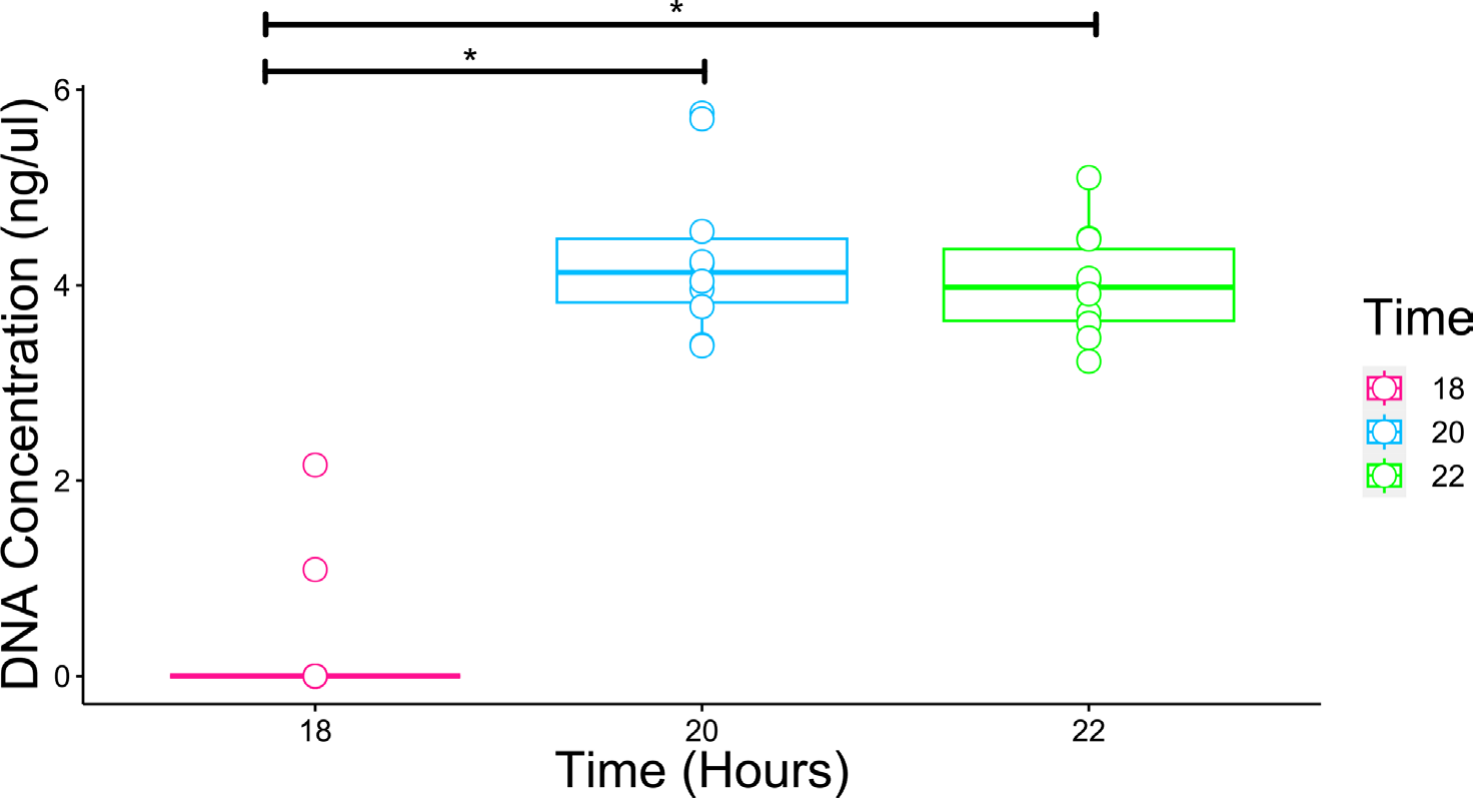
Comparison of DNA yield (ng µl^−1^) from swabs incubated for different periods (*n*=30; 10 swabs per incubation time). The box plots show the average DNA concentrations (ng µl^−1^) for each incubation time. Horizontal bars on each plot show averages, vertical bars show the standard error of the mean (SEM) and lines with an asterisk (*) indicate significant (*P*<0.05) differences.

As some swabs did not yield DNA using method 6, we compared DNA yield from the extracted swabs after different overnight lysis incubation times. Samples were randomly incubated for either 18, 20 or 22 h (Fig. 3). A significant difference between 18 and 20 h and 18 and 22 h (*P*<0.05) was observed, but no significant difference between 20 and 22 h (*P*>0.05). Samples that did not yield detectable amounts of DNA were those incubated for 18 h therefore, future samples were incubated between 20–22 h to obtain higher DNA yield.

#### DNA extracted from skin microbiota is suitable for shotgun metagenomics with short and long read platforms

**Fig. 4.**
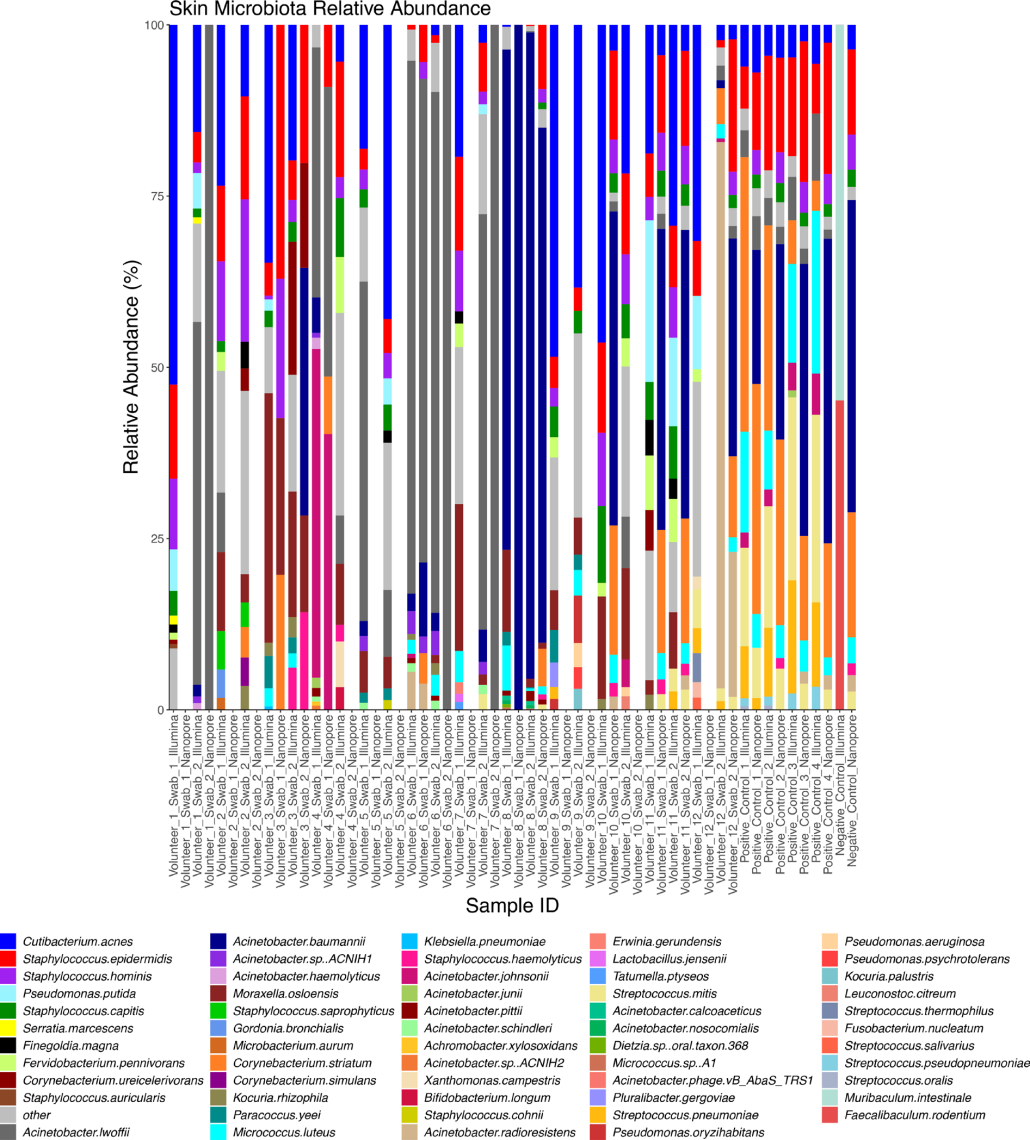
Taxonomic profiles of skin swab microbiota from 12 adult volunteers (two swabs collected from both forearms from each volunteer) generated using Illumina and Nanopore data. Profiles show the relative abundance (percent) of the 10 most abundant species that occur within each sample.

**Fig. 5.**
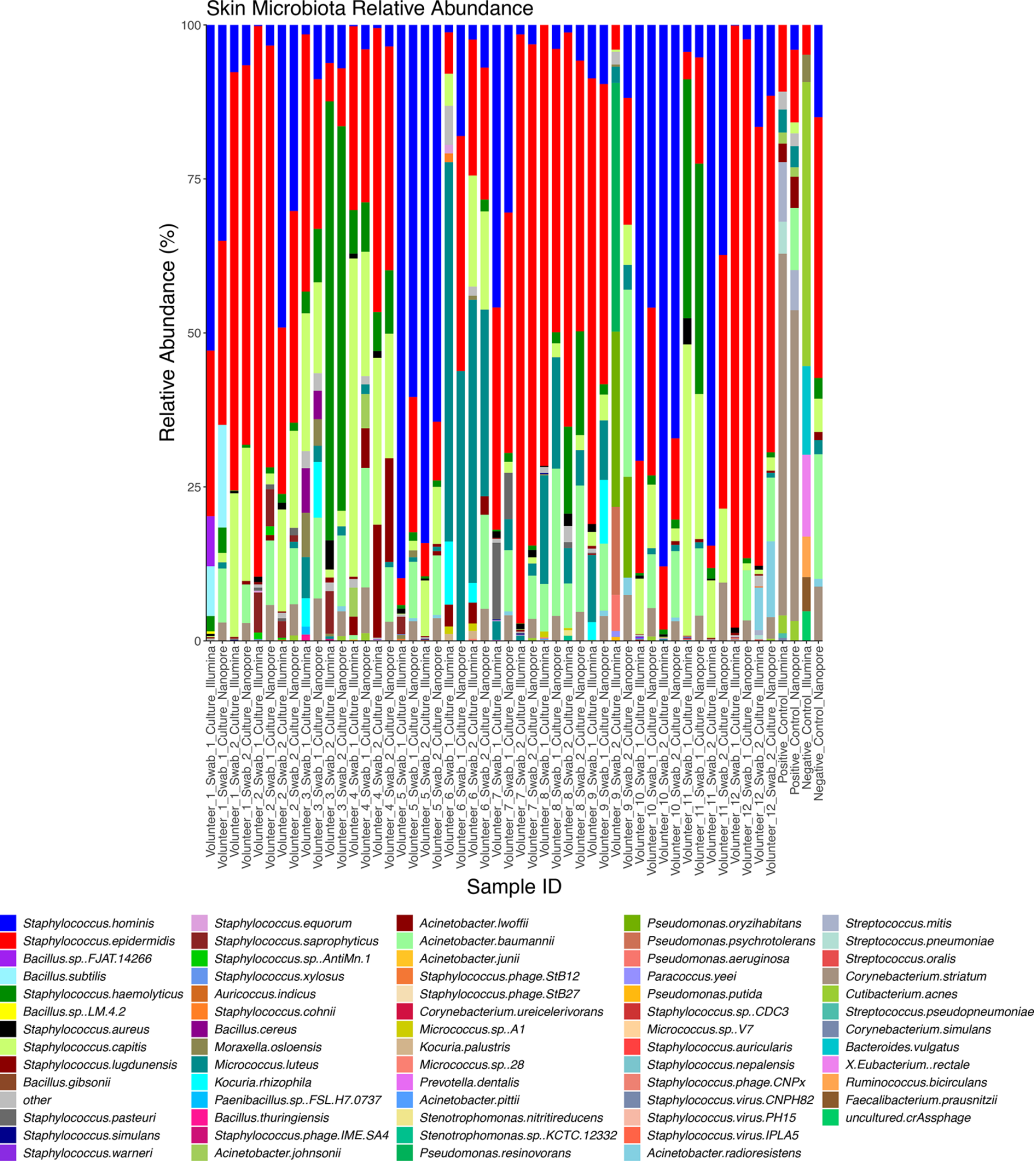
Taxonomic profiles of skin swab culture microbiota from 12 adult volunteers (two swabs collected from both forearms from each volunteer) generated using Illumina and Nanopore data. Profiles show the relative abundance (percent) of the 10 most abundant species that occur within each sample.

**Fig. 6.**
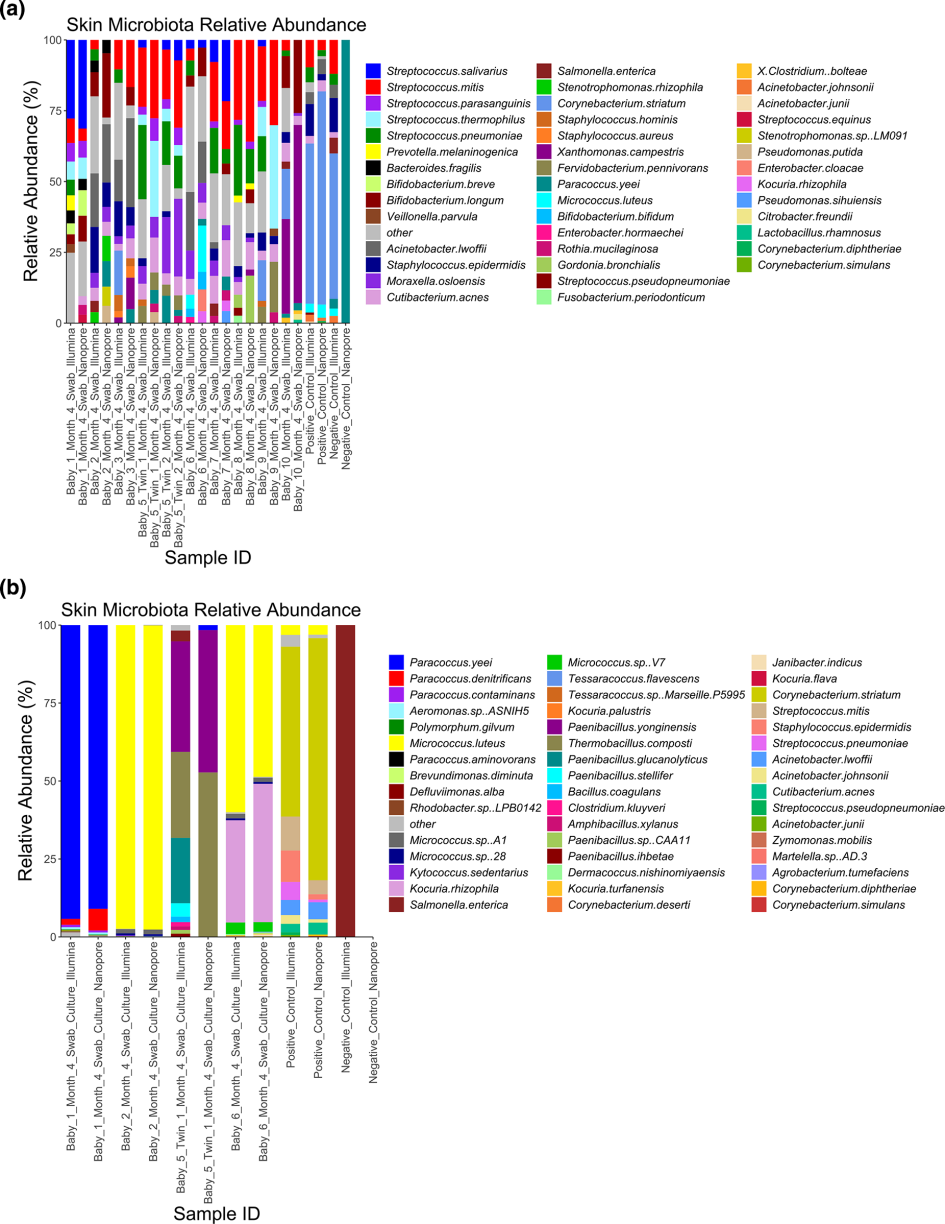
Taxonomic profiles of the skin swab and culture microbiota from ten PEARL babies (one swab collected off one forearm from each baby) generated using Illumina and Nanopore data. Profiles show the relative abundance (percent) of the 10 most abundant species that occur within each sample. (a): Illumina and Nanopore skin swab data. (b): Illumina and Nanopore skin culture data.

After removal of human reads, microbial taxonomic profiles were generated using both Illumina and ONT sequence data using Kraken2 and Bracken (Figs 4–6) swabs and cultures.

The positive controls displayed the expected microbiota from the ATCC skin microbiome whole cell mix – *Acinetobacter johnsonii *, *Corynebacterium striatum *, *Micrococcus luteus *, *Cutibacterium acnes *, *Staphylococcus epidermidis *, *Streptococcus mitis *. There was also a clear reduction in reads from these samples across the dilution series. This demonstrated that the DNA extraction method was able to effectively extract DNA from the diverse range of species present in the ATCC skin microbiome mix. Some background contamination was detected in negative controls, although the number of reads was always much lower than in samples and most DNA fragments identified in the negative controls mapped against organisms not seen in the test samples.

Both Illumina and ONT data indicated a typical skin microbiota from both adult and baby skin swabs and generated enough reads allowing downstream taxonomic analysis at the species level. The adult swabs identified bacteria, viruses, and phages, whereas the baby swabs only displayed bacterial diversity. Baby skin swabs contained more *Streptococcus* and fewer *Staphylococcus* species when compared to adult skin swabs. The baby skin swabs also indicated the presence of *Bifidobacterium longum *, *Bifidobacterium breve* and *Bifidobacterium bifidum *, which are not typical skin residents, but common residents of the infant gut, which likely demonstrates transient skin contamination on the babies [44, 45]. Importantly, data was generated for skin swabs that had very low DNA concentrations. Illumina and ONT platforms identified very similar microbiota profiles for both skin swabs and cultures, with comparable percentage total counts of the most abundant species (those representing more than 0.5 % of each sample) (Fig. S2). Analysis of the taxonomic profile from cultured samples exhibited less microbial diversity than the skin swabs as expected but confirmed the presence of species identified in the SMS. As in the SMS data, adult cultures exhibited more *Staphylococcus* species than *Streptococcus *.

Once a successful DNA extraction method was established, the depth of sequence data required to provide optimal phylogenetic resolution and to construct MAGs were both assessed. This was done by comparing outcomes using 5Gbp per sample and subsamples thereof down to 1Gbp of data. For species identification a rarefaction curve was produced, which showed more species identified as more data was used; though statistical analysis showed there was not a significant difference in species recovery between 2.5 and 5Gbp of data (Fig. S3A). Recovery of MAGS was also higher from samples where 5Gbp of data were used than 1Gbp, although this difference was not found to be statistically significant (Fig. S3B). We were able to isolate MAGs predicted to be from diverse genera showing the method can isolate and assemble DNA from different parts of the phylogeny of the skin microbiota (Table S4). Based on this analysis, 5Gbp of data appears to be adequate for phylogenetic analysis of the skin microbiota using this method, whilst also providing enough data for generation of MAGs and therefore allowing detailed study of the functional capacity of the skin microbiome to be studied based on these results.

## Discussion

We aimed to develop an efficient protocol for DNA extraction suitable for use from both skin swabs and cultured bacterial cells. Initial testing showed both water and PBS were suitable diluents to maintain viability and for DNA extraction in agreement with previous studies [46–48] and PBS was then used throughout. Comparison of a variety of lysis procedures identified the effectiveness of a combined approach using both overnight heated enzymatic (lysozyme) and mechanical (bead beat) lysis methods to result in sufficient DNA yield of a high molecular weight from both Gram-positive and Gram-negative bacteria. Previous work has indicated that the type of enzyme and mechanical intensity is also important for lysis of different bacterial species [49–51]; however, our combined use of a mechanical and enzymatic lysis approach resulted in an unbiased extraction of Gram-positive and Gram-negative bacteria, which was validated by the production of expected profiles from the positive control mock community [52]. We used the Maxwell DNA isolation platform which was able to reliably produce DNA of good quality and molecular weight suitable for SMS. Other platforms are available and likely to work well with this method, we did not seek to benchmark extraction kits, but this could be done in future to refine this method further.

Given the low biomass of skin microbiota, some of the adult skin swabs produced very low/absent DNA concentrations and paired cultures also indicated low bacterial burden. Individual variations when swabbing (pressure, direction, frequency) can affect the yield of DNA and viable bacteria, and are difficult to control [9] and may be responsible for this variation. A swabbing method was used as it is commonly used to collect skin microbiome samples [9] and was already used by our local PEARL study to collect samples due to its non-invasive nature, which is suitable for neonates, who have an underdeveloped skin structure [53, 54]. We also found a difference in sensitivity between platforms for samples with low amounts of DNA, some adult swabs did not produce data using the ONT platform although these same samples generated bacterial cultures. As the ONT platform requires more input DNA to generate data than Illumina platforms [16], the inability to generate data for some samples was not surprising as skin swabs can be low biomass [14, 15]. However, increasing the overnight incubation time did improve DNA yield, and the Illumina sequencing resulted in generated data for all samples.

Most samples did generate data from both Illumina and ONT platforms which presented similar microbiota profiles from skin swabs and cultures. Typical adult skin microbiota (Phyla; *Pseudomonadota *, *Actinomycetota *, and *Bacillota *) [2, 55, 56] and infant skin microbiota (Phyla; *Bacillota *, *Actinomycetota *, *Pseudomonadota *, and *Bacteroidota *) [57] were detected. We focused on bacterial species identified, but the protocol did identify other skin microbiota (viruses, phages and fungi), although only from adult volunteers [55]. Other researchers can use this protocol as a starting point to be adapted if these organisms are their focus. Baby profiles only contained bacteria, and demonstrated less microbial diversity than adults, which has been shown in previous studies [58]. Baby skin did exhibit more *Streptococcus* species than adult skin, which agrees with previous work demonstrating a predominance of *Streptococcus* species in early age, which decreases with age [57, 58]. Interestingly, sequencing of swabs from infant skin identified *Bifidobacterium* species, which are not typical skin residents, but rather maternal and infant gut residents and they can also be found in breast milk [44, 45]. Given the paired cultures did not result in any *Bifidobacterium* isolates, this is likely to indicate transient transfer to the babies’ skin through breast feeding. The babies with available metadata that showed *Bifidobacterium* presence on the skin were all breast fed at some point between birth and month 4.

Whilst skin is a relatively low biomass environment, we did not need to include any methods to mechanically deplete human DNA or selectively enrich microbial DNA before SMS [59], which have been needed in some other studies on low biomass samples. These enrichment approaches do not reliably target all species [59], can skew the resulting genomic profiles [60] and depletion can result in some loss of bacteria [59], thus further steps are required for downstream analysis. In our described method, we generated enough data, and depleted human DNA computationally, therefore precluding the need for any additional steps that may introduce biases and skew skin microbiota profiles.

Both Illumina and ONT sequence data allowed identification of all ATCC positive control species, with a clear reduction in read number across the dilution series. These results further demonstrate the effectiveness of the extraction method and utility of both sequencing platforms. Inclusion of a commercially available mixed community positive control, with a known cell concentration, is important for standardising the extraction process, and serial diluting the positive control can determine the limit of detection [61]. This is also helpful when comparing different sequencing runs and sample sets, allowing more robust comparisons to be made. Although, we tried to define a limit of detection for DNA concentration and read number required for effective SMS, we had several swab samples that did not obtain a DNA concentration reading, but usable reads were produced for taxonomic profiling. Therefore, no obvious cut-off for a limit of detection was determined, and indeed there is also no ‘defined’ limit identified in the literature for low biomass samples, such as skin swabs.

We did identify some background contamination in the negative controls, contamination commonly occurs in metagenomic studies, especially those with low biomass samples [62]. Several studies have identified contamination sources occurring from neighbouring samples and the ‘kitome’ [62, 63]. Contamination within a dataset can be identified and removed using bioinformatic techniques [64, 65] although low biomass samples have a higher risk of true microbial microbiota members being removed [66]. Given the background contaminants in the controls were at a very low level and mostly represented species not seen in the test samples, we did not remove them as they had a negligible impact on the profiles produced.

We determined that the generation of 5Gbp of Illumina data from a skin swab was suitable for microbial species profiling and produced a limited number of MAGs. MAGs are important for in-depth functional information [13] and indicate genome quality [39, 67, 68], and they can be used to identify novel taxa and allow further comparison with whole genome sequence data from isolates. Our method is compatible with both Illumina and ONT platforms, and combining a higher sequencing depth with ONT, the data has potential to improve the number and quality of MAGs to be recovered [13, 69, 70].

## Conclusion

An optimised medium-throughput DNA extraction, SMS, and analysis approach can effectively characterise the skin microbiota from adults and babies. This method can be applied for in-depth analysis of cohort studies allowing identification of taxonomic and functional changes of mothers and infants over time and should allow comparison to other body sites (e.g. the gut). Robust microbiota profiling, particularly in less well studied niches such as the skin, is important for the development of methods to alter microbiome compositions for health.

## Supplementary Data

Supplementary material 1
