# Supplementary material for: An efficient method for high molecular weight bacterial DNA extraction suitable for shotgun metagenomics from skin swabs
Source: Microb Genom. 2023 Jul 10;9(7):mgen001058. doi: 10.1099/mgen.0.001058 (PMC10438817; doi:10.1099/mgen.0.001058)
Supplement: Supplementary material 1 [file mgen-9-1058-s001.pdf]

Supplementary File 1: The Promega Maxwell® RSC 48 Instrument and RSC Blood DNA Kit Protocol

1. Mix all blood samples for at least 5 minutes at room temperature.
2. Prepare and label incubation tubes compatible with a heating block.
3. Add 30µl of Proteinase K (PK) Solution to each incubation tube.
4. Add liquid blood (up to 300µl) to each incubation tube.
5. Add 300µl of Lysis Buffer to each incubation tube.
6. Vortex each tube for 10 seconds.
7. Incubate each tube in the heating block (set to 56°C) for 20 minutes. During this incubation, prepare cartridges and turn on the Maxwell where you will proceed through a self-check. Change gloves before handling Maxwell® RSC cartridges, RSC plungers and elution tubes (0.5ml).
8. Select the RSC Blood DNA method, then select the cartridge positions to be used for this extraction run. The door should automatically open to load samples.
9. For each cartridge, vortex the well containing beads to resuspend them, then prepare the cartridges on the cartridge rack.
10. Place each cartridge in the rack with the label identifier side facing away from the 0.5ml elution tubes. Press down on the cartridge to snap it into position. Carefully peel back the seal so that all plastic comes off the top of the cartridge - ensure that all sealing tape and any residual adhesive are removed.
11. Add 50µl of Elution Buffer to the bottom of each elution tube and place into the elution tube position for each cartridge.
12. Transfer each blood lysate sample from the incubation tube to well #1 of each cartridge; Well #1 is the well closest to the printed side and furthest from the elution tub.
13. Place one plunger into well 8 of each cartridge.
14. Transfer the Maxwell® RSC cartridge rack containing the prepared cartridges on the Maxwell® RSC 48 instrument platform. Ensure that the cartridge rack is level on the instrument platform.
15. Press the Run/Stop button. The platform will retract. Close the door.
16. The Maxwell® RSC 48 instrument will immediately begin the purification run. The screen will display the steps performed and the approximate time remaining in the run.
17. When the automated purification run is complete, the LCD screen will display a message that the method has ended.

18. Follow on-screen instructions at the end of the method to open door.
19. Verify that plungers are located in well 8 of the cartridges at the end of the run. If plungers are not removed from the magnetic plunger bar automatically, push them down gently by hand to remove them.
20. Remove the Maxwell® RSC Cartridge Rack from the instrument and dispose in the sharps bin, remove elution tubes containing DNA and cap the tubes.

For full protocol and technical details on setting up the Maxwell instrument, follow the Promega technical Maxwell RSC Blood DNA kit AS1400 and ASB1400 TM419 manual.

Supplementary File 2: PEARL study swabbing instructions:

1. To perform swabbing, pull and twist the swab out of its tube taking care not to touch the tip (swab end) with anything other than the area to be swabbed.
2. Holding the swab in one hand, roll the opposite end (swab end) along the inside of the forearm from the wrist to the elbow pit ensuring that the whole of the swab end has come in to contact with the volunteer participant skin.
3. Replace the swab back into the tube ensuring the lid is on tight.
4. The swab may leave a grey residue on the forearm. Once sampling is complete, wash the area with soap and water.
5. The date of sample collection and Biorepository sample ID will be written on the container in pen.
6. Samples will be stored in a 4°C fridge until samples are collected for testing.

Supplementary File 3: Processing, culturing, and extracting bacterial DNA from Skin Swab Samples using the Promega Maxwell® RSC 48 instrument and RSC Blood DNA kit

Processing skin swabs for DNA extraction, culturing, and preparing glycerol stocks:

1. Appropriately label one 1.5ml Eppendorf tube for each swab.
2. Prepare the appropriate volume of 1x PBS from the 10x PBS stock solution using Ultrapure Milli-Q water. Add 1ml of 1x PBS to the labelled 1.5ml Eppendorf tubes for each swab.
3. For each run use a negative control containing PBS to ensure no contamination has occurred and use the ATCC Skin Microbiome Whole Cell Mix as a positive control, which is an established mock community, to demonstrate that any negative results are a lack of material rather than an extraction problem.
4. Prepare the ATCC Skin Microbiome Whole Cell Mix using the manufacturer instructions (Skin Microbiome Whole Cell Mix - MSA-2005):
   a. Add 1 mL of chilled 1x PBS to the lyophilized pellet (chill the PBS in the fridge before beginning).
   b. Allow the pellet to dissolve for a minimum of two minutes; keep the vial on ice to prevent cell lysis (this vial contains $1.2x10^{-8}$ cells).
   c. Mix the suspension gently by pipetting up and down a few times and aliquot 200µL into five Eppendorf tubes (to work out the number of cells in each of the five vials, divide $1.2x10^{-8}$ by 5 = 24,000,000 cells).
   d. Centrifuge the tubes for 10 min at 10,000 x g at 4°C and carefully discard the supernatant, then freeze the pellets in the -20°C freezer for future use; Each pellet should contain 24,000,000 cells.
5. Resuspend one of the frozen pellets with 1ml of chilled 1x PBS; If dilutions of the positive control are required, resuspend the frozen pellet with 200µl of 1x chilled PBS, then prepare dilutions up to 10^{-3} and work out the number of cells in the dilutions:
   a. No. Cells in 10^{-1}: 24,000,000÷10 = 2,400,000 cells
   b. No. Cells in 10^{-2} = 24,000,000÷100 = 240,000 cells
   c. No. Cells in 10^{-3} = 24,000,000÷1000 = 24,000 cells
6. Process the swabs by cutting each swab head, using a disposable, sterile scalpel, into a 1.5ml Eppendorf tube containing 1ml of 1x PBS; If positive control dilutions were made, top up the positive controls to 1ml using 1x PBS.
7. Vortex the tubes @ full speed for 2 minutes.
8. Centrifuge tubes at 14,000 x g for 15 minutes to pellet the cells and remove the supernatant.
9. Resuspend the cells in 500µl of 1x PBS.
10. Appropriately label two Colombia Blood Agar plates for each swab (one plate for anaerobic culturing and one plate for aerobic culturing).

11. Pipette 50µl onto the labelled aerobic agar plate, and 50µl onto the paired anaerobic plate.
12. Incubate the aerobic plate in the 37° incubator and the anaerobic plate in the 37° anaerobic cabinet for 48-72 hours.
13. From the remaining 400µl of suspension, pipette 100µl into 1ml of 40% glycerol, prefilled in a 2ml cryovial, to form a glycerol stock of the original skin cells. Freeze the glycerol stock in the -80°C freezer to cryopreserve the skin cells.
14. Centrifuge the remaining 300µl of suspension at 14,000 x g for 15 minutes to pellet the cells; remove the supernatant and keep the pellet for DNA extraction. Proceed to section **Skin swab DNA extraction procedure using the Maxwell Blood DNA kit** (Page 2) to begin the DNA extraction procedure.
15. After the 48h incubation, combine all colonies from the paired aerobic and anaerobic agar plates into a glycerol stock to cryopreserve the cultured skin microbiome. This can be performed by aseptically picking all colonies from both agar plates using a 10µl loop and inoculating them into 1ml of 40% glycerol, prefilled in a 2ml cryovial. Freeze the glycerol stock in the -80°C freezer.
16. A DNA extraction should also be performed on these cultures following section **Skin swab culture DNA extraction procedure using the Maxwell Blood DNA kit** (Page 4).

Skin swab DNA extraction procedure using the Maxwell Blood DNA kit:

1. Resuspend the swab pellet in 400µl of 1x PBS and transfer the 400µl of sample with the original swab head into a 2ml Lysing Matrix E tube, then add 3µl of the Thermo Fischer Lysozyme (dilute to 250U/µl with Tris- EDTA Buffer (Sigma-Aldrich)); For ease of use prepare a 300 µl working stock of the lysozyme at 250U/µl by adding 75µl of the Lysozyme to 225µl of Tris-EDTA Buffer(Sigma-Aldrich)) and incubate with agitation at 300rpm, 37oC for 20-22 hours.
2. Following incubation, perform a bead beating step using the Qiagen Tissue Lyser instrument for 3 minutes at 20 Hz followed by an off-board lysis.
3. Off-board lysis is performed by adding 40 µl proteinase K, 165 µl Buffer ATL, 120 µl Carrier RNA (resuscitate each vial containing 310µg of lyophilised Carrier RNA with 310µl of Buffer AVE to make a 1µg/µl solution (instructions from the QIAMP® Viral RNA Mini Handbook)), and 315 µl Buffer ACL (all offboard-lysis reagents are from Qiagen) into the 2ml Lysing Matrix E tubes (MP Biomedicals) containing 400ul of sample and incubate the samples at 68 °C for 15 minutes on a heat block (the off-board lysis protocol was obtained from the QIASymphony SP Protocol Sheet - Complex400 OBL V4 DSP Virus Bacteria).
4. Centrifuge samples at 14,000 x g for 15 minutes to pellet the cells.
5. Whilst the samples are centrifuging, label the elution tubes (provided with the Promega RSC Blood DNA kit) with Sample IDs and add 50µl of Elution Buffer (provided with the Promega

RSC Blood DNA kit) to the bottom of each 0.5ml elution tube; Change gloves before handling Maxwell® RSC cartridges, RSC plungers and elution tubes (provided with the Promega RSC Blood DNA kit).

6. Turn on the Maxwell® RSC 48 Instrument and proceed through a self-check.
7. Select the RSC Blood DNA method, then select the cartridge positions to be used for this extraction run and the door should automatically open.
8. For each cartridge, vortex the well containing beads to resuspend them, then prepare the cartridges on the cartridge rack (Refer to Figure 1).
9. Remove the cartridge rack from the Maxwell instrument and prepare cartridges in the rack; Place each cartridge in the rack with the label identifier side facing away from the 0.5ml elution tubes (Refer to Figure 1); The cartridges will only fit in the instrument in this orientation. Press down on the cartridge to snap it into position. Carefully peel back the seal so that all plastic comes off the top of the cartridge - ensure that all sealing tape and any residual adhesive are removed.
10. After the samples have been centrifuged, add the supernatant to well #1 of each cartridge and place one plunger into well #8 of each cartridge (Note: Once the supernatant is added to well #1, also hold the pipette tip onto the swab present in the tube and take up any supernatant that the swab has soaked up to reduce loss of genetic material); Well #1 is the well closest to the label identifier side, and well #8 is closest to the elution tube (Refer to Figure 1).
11. Transfer the prepared cartridges in the rack to the Maxwell® RSC 48 instrument platform. Ensure that the cartridge rack is level on the instrument platform.
12. Place elution tubes containing the Elution Buffer into the elution tube position for each cartridge (with the elution tube lids open, facing away from the cartridge - Refer to Figure 1).

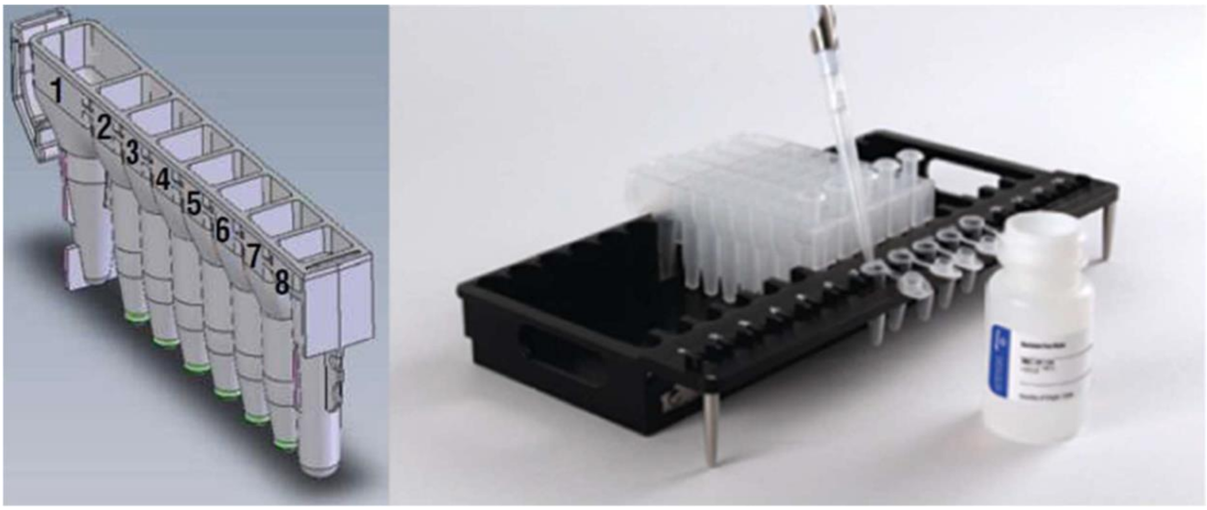

Figure 1 – Preparing Cartridges and Elution Tubes on the Cartridge Rack (Obtained from the Promega technical Maxwell RSC Blood DNA kit AS1400 and ASB1400 TM419 manual).

13. Press the Run/Stop button. The platform will retract, and the door will close.
14. The Maxwell® RSC 48 instrument will immediately begin the purification run. The screen will display the steps performed and the approximate time remaining in the run.
15. When the automated purification run is complete, the LCD screen will display a message that the run has ended.
16. Follow on-screen instructions at the end of the run to open the door.
17. Verify that the plungers are located in well #8 of the cartridges at the end of the run. If plungers are not removed from the magnetic plunger bar, push them down gently by hand to remove them.
18. Remove elution tubes containing DNA and cap the tubes.
19. Remove the Maxwell® RSC cartridge rack from the instrument and dispose of the cartridges into the Maxwell waste sharps bin.
20. DNA extracts can now be used for downstream use.

Skin Swab Culture DNA Extraction Procedure Using the Maxwell Blood DNA Kit:

1. Appropriately label one 1.5ml Eppendorf tube for each culture.
2. Prepare the appropriate volume of 1x PBS from the 10x PBS stock solution using Ultrapure Milli-Q water. Add 800µL of the 1x PBS to the labelled 1.5ml Eppendorf tubes.
3. Add 1ml of 1x PBS to a 1.5ml Eppendorf tube for the Negative control and 1ml of chilled 1x PBS to one of the Eppendorf tubes containing the previously frozen ATCC Skin Microbiome Whole Cell Mix pellet, as described above in step 5 under section **Processing Skin Swabs for DNA Extraction and Preparing Glycerol Stocks** (Page 1).
4. For each run a negative control containing PBS must be used to ensure no contamination has occurred and the ATCC Skin Microbiome Whole Cell Mix must be used as a positive control, which is an established mock community, to demonstrate that any negative results are a lack of material rather than an extraction problem.
5. Thaw the culture glycerol stocks on ice, pipette up and down a few times to mix the suspension, and add 200µL of the stocks to the appropriately labelled tubes containing 800µL of the 1x PBS, to add up to 1ml.
6. Vortex the tubes @ full speed for 2 minutes.
7. Centrifuge the tubes at 14,000 x g for 15 minutes to pellet the cells and remove the supernatant.
8. Resuspend the culture pellet in 400µl of 1x PBS and transfer the 400µl of sample into a 2ml Lysing Matrix E tube, then add 3µl of the Thermo Fischer Lysozyme (dilute to 250U/µl with Tris-EDTA Buffer (Sigma-Aldrich); For ease of use prepare a 300 µl working stock of the lysozyme at 250U/µl by adding 75µl of the Lysozyme to 225µl of Tris-EDTA Buffer (Sigma-Aldrich)) and incubate with agitation at 300rpm, 37oC for 20-22 hours.

9. Following incubation, perform a bead beating step using the Qiagen Tissue Lyser instrument for 3 minutes at 20 Hz followed by an off-board lysis.
10. Off-board lysis is performed by adding 40 µl proteinase K, 165 µl Buffer ATL, 120 µl Carrier RNA (resuscitate each vial containing 310µg of lyophilised Carrier RNA with 310µl of Buffer AVE to make a 1µg/µl solution (instructions from the QIAMP® Viral RNA Mini Handbook)), and 315 µl Buffer ACL (all offboard-lysis reagents are from Qiagen) into the 2ml Lysing Matrix E tubes (MP Biomedicals) containing 400ul of sample and incubate the samples at 68 °C for 15 minutes (the off-board lysis protocol was obtained from the QIASymphony SP Protocol Sheet - Complex400 OBL V4 DSP Virus Bacteria).
11. Centrifuge the samples at 14,000 x g for 15 minutes to pellet the cells.
12. Follow steps 5-20 under section **Skin Swab DNA Extraction Procedure Using the Maxwell Blood DNA kit** (Pages 2-4).

Supplementary File 4: Shotgun Metagenomic Sequencing Using Illumina

The QIB sequencing team used the tagmentation based library construction using the Illumina DNA Prep Kit (Illumina Catalogue No. 20018704) because it is cost-effective and provides a better representation of the sample contents. These findings resulted from an experiment comparing the Illumina DNA Prep kit with other library construction methods - Nextera XT, KAPA HyperPlus, NEBNext Ultra II, QIAseq FX, TruSeq nano, KAPA HP PCR-free workflow and TruSeq DNA PCR-free.

Where possible, samples were normalised to 5ng/µl with PCR-grade water. Some of the skin swab DNA Qubit concentrations were lower than 5ng/µl and therefore could not be normalised to 5ng/µl. A bead tagmentation of metagenomic DNA was then performed whereby transposomes are randomly inserted into the genomic DNA adding Illumina adapters enabling barcoding and amplification of the libraries for multiplexing on an Illumina run. This tagmentation was a reduced reaction (20-fold dilution) and was prepared by mixing 0.5 µl of Tagmentation buffer (TB1), 0.5 µl Bead Linked Transposomes (BLT)(Illumina Catalogue No. 20018704) and 4 µl PCR grade water. 5µl of this master mix was then added to a 96 well plate. 2µl of normalised DNA (10ng total) was pipette mixed with the 5µl of the tagmentation mix and heated to 55 ºC for 15 minutes in a PCR block. For samples with less than 5ng/µl, a separate tagmentation master mix was used with no water added and instead 6µl input DNA volume. The tagmented samples were then barcoded by setting up the following PCR master mix: 10µl KAPA 2G Fast Hot Start Ready Mix (Merck Catalogue No. KK5601) and 2µl PCR grade water per sample. 12µl of the master mix was added to each well in a 96-well plate. 1µl of 10µM custom made primer stock containing both P7 and P5 Illumina 9bp barcodes (Illumina Catalogue No. FC-131-2001 to 2004) were added to each well. Finally, the 7µl of the tagmentation mix was added and mixed. The PCR was run with 72ºC for 3 minutes, 95ºC for 1 minute, 14 cycles of 95ºC for 10s, 55ºC for 20s and 72ºC for 3 minutes.

The individual libraries were quantified using the Promega QuantiFluor® dsDNA System (Catalogue No. E2670) and run on a GloMax® Discover Microplate Reader and run on the Agilent Tapestation 4200 instrument using a D5000 screentape (Agilent Catalogue No. 5067-5579) to check the library size distribution. Libraries were pooled based on ng/µl and the pooled library was double-SPRI size selected between 0.5 and 0.7X bead volumes using sample purification beads supplied in the Illumina DNA prep kit (Illumina® DNA Prep, (M) Tagmentation (96 Samples, IPB), 20060059). The final Illumina pooled library was then quantified using the Qubit 3.0 fluorometer and run on the Agilent 4200 Tapestation instrument. Both the qubit concentration and average insert size (determined by the tapestation) were used to estimate the molarity of the final pool. The final pool was denatured and diluted to 1.5pM and loaded on the NextSeq500 Instrument using a Mid-output 300 cycle kit (Illumina Catalogue FC-404-2003) with paired-end 150bp chemistry. The Illumina recommended

denaturation and loading recommendations were followed, which included a 1% PhiX spike in (PhiX Control v3 Illumina Catalogue FC-110-3001). The sequencing team aimed for and obtained approximately 300Mbp data per sample. The Bioinformatics team at QIB converted the raw data into 8 FASTQ files for each sample. These were then run through FastP to pre-process the FASTQ files and remove the Illumina adapters.

Shotgun Metagenomic Sequencing Using Oxford Nanopore

Oxford Nanopore libraries were prepared using a novel modified Illumina DNA Prep ultra-cost-effective method (Illumina® DNA Prep, Tagmentation 20018704). Primers were designed with a 3' end compatible with the Nextera transposon insert, a 24bp barcode at the 5' end and included a pad and spacer. The same barcode was used at each end using up to 96 combinations. Primer pairs were mixed at a concentration of 10µM. The tagmentation reaction as described above was set up. Where possible, DNA was normalised to 50ng/µl and 2µl were used in the tagmentation reaction. For low biomass samples that could not be normalised, 6µl DNA was used and the water was omitted from the master mix. A PCR master mix was prepared using 10µl NEB LongAmp Taq 2x Master Mix (Catalog no. M0287S) and 2µl PCR grade water per sample. 12µl was added to each well. 1µl of the appropriate primer pair at 10µM was added to each well. Finally, the 7µl of the tagmentation mix was added and mixed. The PCR was run with 94ºC for 1 minute followed by 14 cycles of 94ºC for 30 seconds, 55ºC for 1 minute and 65ºC for 30 minutes.

Following the PCR reaction, libraries were quantified using the Promega Quantifluor ds DNA system (Catalogue No. E2670) and run on a Promega Glomax plate reader. Libraries were pooled together in equal nanograms and the SPRI size was selected at 0.5X bead volume using sample purification beads (Illumina® DNA Prep, (M) Tagmentation (96 Samples, IPB), 20060059) with final elution in 30µl. The pooled library was prepared for sequencing on a MinION flow cell (R9.4.1) following the Nanopore SQK-LSK109 protocol with a few modifications. The DNA end-repair reaction was extended to 15 mins, omitted the FFPE repair buffer and enzyme mix and instead used 7µl Ultra II End-prep buffer and 3µl Ultra II End-prep enzyme mix (cat no. E7546L). The library was loaded on the minION in accordance with manufacturer's instructions. The data coverage varied from 300Mbp to 10Gbp per sample. The Bioinformatics team at QIB converted the raw data into FASTQ files using their customised guppy method. FASTQ files were run through FastP to pre-process the samples and remove adaptors.

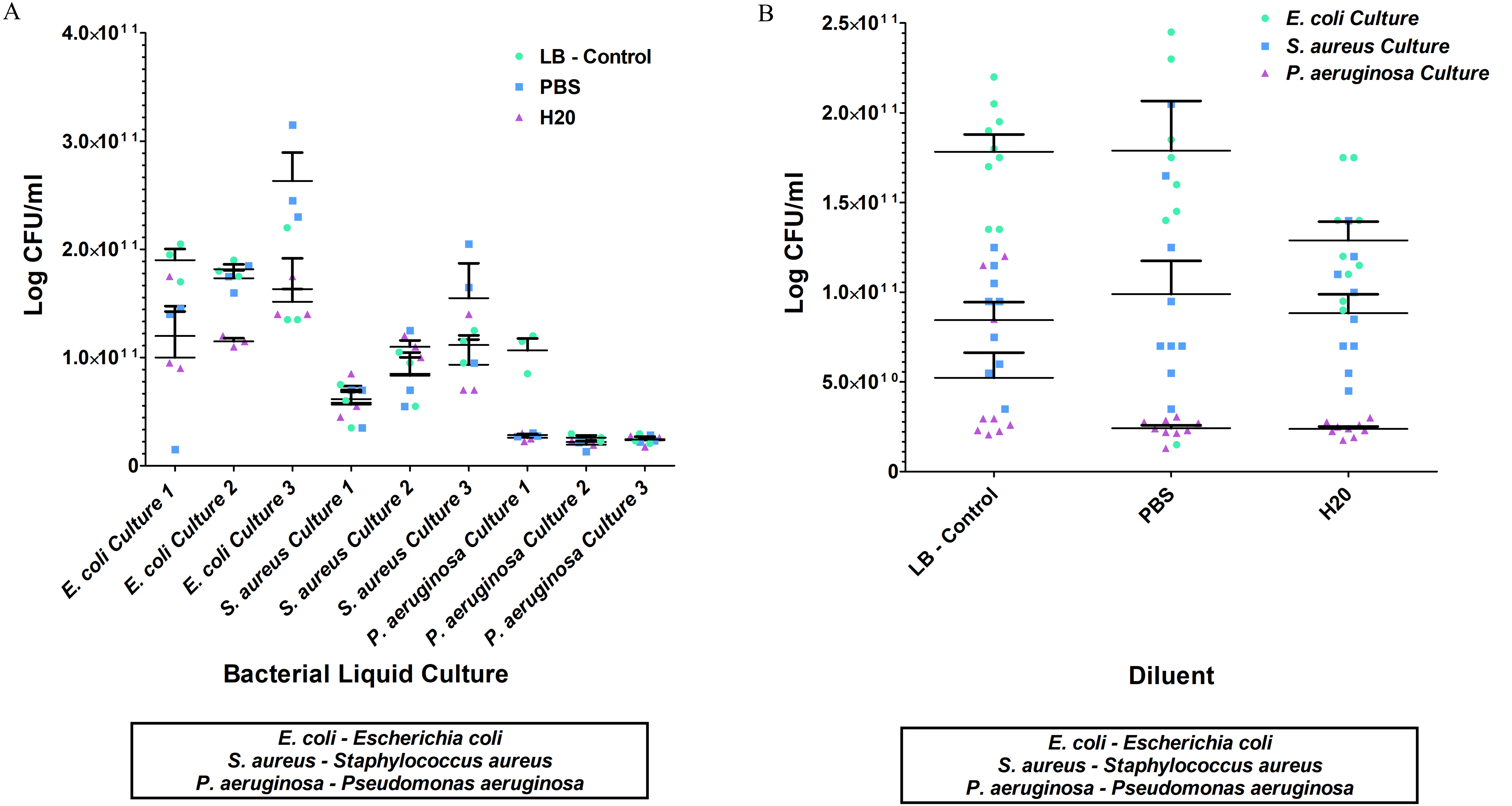

Figure S1 - Bacterial growth from cultures for *Escherichia coli*, *Staphylococcus aureus* and *Pseudomonas aeruginosa* that were processed and grown in 1x PBS and Milli-Q water. A and B: CFU/ml recovered for each isolate replicate cultured in LB broth, 1x PBS and Milli-Q water, and the reproducibility of replicates in each diluent condition (n=81; nine replicates for each species per diluent). The horizontal bars on each plot show the average and vertical lines show the SEM.

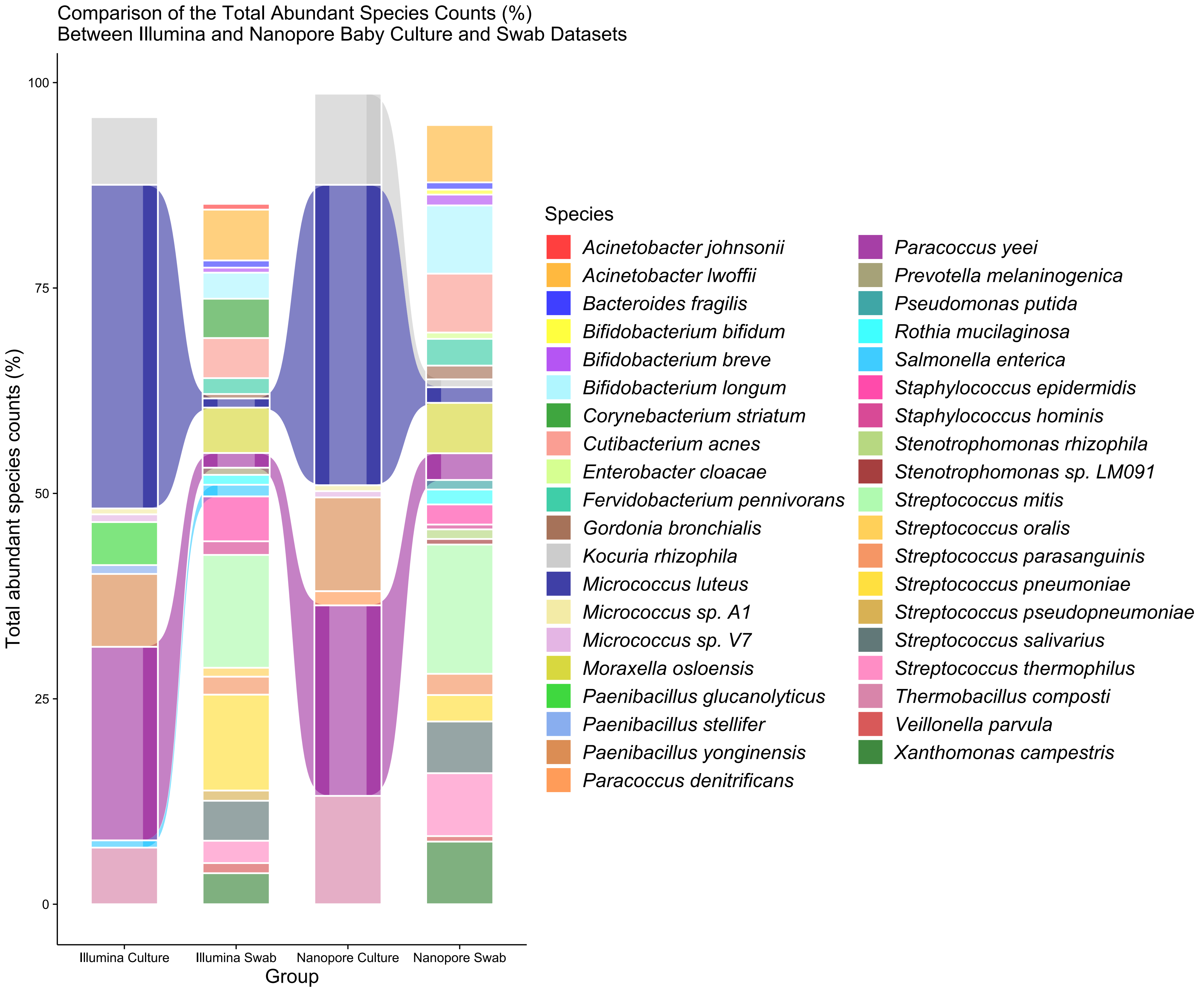

Figure S2 - Alluvial plot of the percentage total abundant species counts between Illumina and Nanopore SMS baby skin swab and culture datasets (one swab collected off one forearm from ten babies aged four months old). This was calculated by adding the total counts of each species, which were converted into a percentage of the total species count; only species with >0.5% of counts were used. The adjoining lines show the abundant skin species that were detected by both platforms.

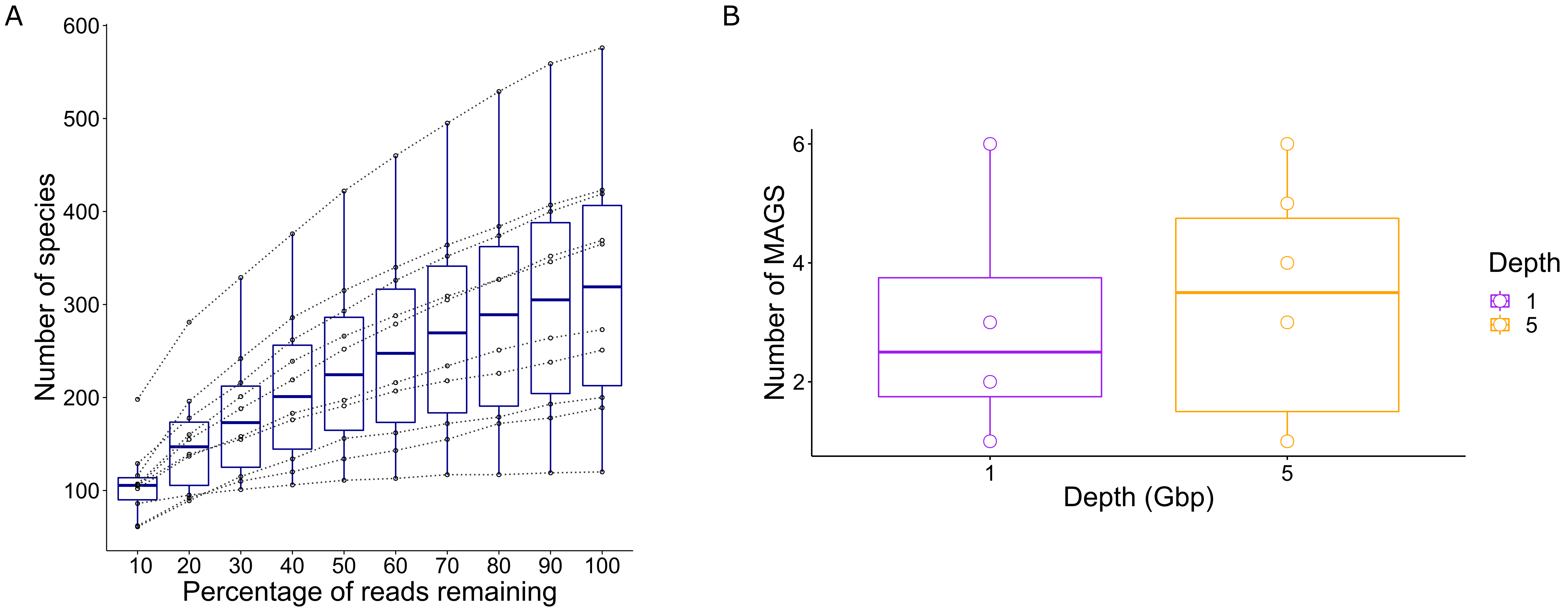

Figure S3 - Species and MAGs recovery from ten PEARL baby skin swabs (collected off one forearm from each baby aged four months old). A: Species recovery at 5Gbp sequencing depth and subsampled depths. The horizontal bars give the average number of species recovered at each level of reads, and the dots and lines show how each sample changes with reduced sequencing depth. 10%-100% reads represents 0.5Gbp-5Gbp at increments of 0.5Gbp. B: MAGs recovery at 1Gbp and 5Gbp sequencing depths. The horizontal bars give the average number of MAGs at each sequencing depth; the key to the right of the plot has colour coded the depth, with 1Gbp in purple and 5Gbp in orange. The vertical lines on each box plot show the SEM.

Table S1 – PEARL study baby metadata

Sample ID	Age	Sex	Health status at birth	Breast fed at birth	Feed at week 3	Feed at month 4
Baby 1	Month 4	Female	Healthy	No	Missing Data	Missing Data
Baby 2	Month 4	Female	Healthy	No	Missing Data	Formula
Baby 3	Month 4	Male	Healthy	Yes	Breast	Breast
Baby 5 twin 1	Month 4	Female	Healthy	No	Breast/formula	Breast/formula
Baby 5 twin 2	Month 4	Male	Healthy	No	Breast/formula	Breast/formula
Baby 6	Month 4	Male	Healthy	Yes	Breast	Breast
Baby 7	Month 4	Female	Healthy	Yes	Breast	Breast
Baby 8	Month 4	Male	Healthy	Yes	Breast/formula	Formula
Baby 9	Month 4	Male	Healthy	No	Missing Data	Missing Data
Baby 10	Month 4	Male	Healthy	Breast/formula	Breast/formula	Formula

Table S2 – Bacterial DNA concentrations from the six DNA extraction methods

Overnight Liquid Culture	Method 1 BR Qubit (ng/µl)	Method 2 BR Qubit (ng/µl)	Method 3 BR Qubit (ng/µl)	Method 4 BR Qubit (ng/µl)	Method 5 BR Qubit (ng/µl)	Method 6 BR Qubit (ng/µl)
E. coli - Culture 1	19.9	11.5	36.8	47.2	40.9	46.8
E. coli - Culture 2	33.2	11.3	46.5	55.3	54.9	38.5
P. aeruginosa - Culture 1	8.68	18.6	63.9	30.8	88.4	96.7
P. aeruginosa - Culture 2	7.96	12.2	33.5	42.7	97.7	104
S. aureus – Culture 1	9.7	5.18	32.8	29.7	51.2	37.8
S. aureus – Culture 2	8.76	4.16	28.7	39.7	49.8	45.6

Table S3 – DNA concentrations of skin swabs

Sample ID	HS Qubit (ng/µl)
Volunteer 1 – Swab 1	< 0.50
Volunteer 1 – Swab 2	< 0.50
Volunteer 2 – Swab 1	< 0.50
Volunteer 2 – Swab 2	< 0.50
Volunteer 3 – Swab 1	< 0.50
Volunteer 3 – Swab 2	< 0.50
Volunteer 4 – Swab 1	< 0.50
Volunteer 4 – Swab 2	< 0.50
Volunteer 5 – Swab 1	1.09
Volunteer 5 – Swab 2	2.16
Volunteer 6 – Swab 1	3.31
Volunteer 6 – Swab 2	1.67
Volunteer 7 – Swab 1	0.68
Volunteer 7 – Swab 2	0.477
Volunteer 8 – Swab 1	< 0.50
Volunteer 8 – Swab 2	0.156
Volunteer 9 – Swab 1	0.837
Volunteer 9 – Swab 2	0.809
Volunteer 10 – Swab 1	6.52
Volunteer 10 – Swab 2	9.73
Volunteer 11 – Swab 1	10.5
Volunteer 11 – Swab 2	5
Volunteer 12 – Swab 1	1.69
Volunteer 12 – Swab 2	3.63
Positive Control 1	2.47
Positive Control 2	1.1
Positive Control 3	0.41
Positive Control 4	0.261
Negative Control	0.064
Baby 1 – Month 4 – Swab	5.1
Baby 2 – Month 4 – Swab	4.05
Baby 3 – Month 4 – Swab	4.48
Baby 5 – Twin 1 – Month 4 – Swab	4.07
Baby 5 – Twin 2 – Month 4 – Swab	3.71
Baby 6 – Month 4 – Swab	4.47
Baby 7 – Month 4 – Swab	3.61
Baby 8 – Month 4 – Swab	3.91
Baby 9 – Month 4 – Swab	3.22
Baby 10 – Month 4 – Swab	3.46
Positive Control (ATCC community)	4.53
Negative Control (PBS)	2.69

Table S4 – Recovery of MAGS from skin microbiota

Sample	Number of independent MAGs recovered	Predicted genus of novel MAGs
E005-1-B	3	*Paracoccus*
		Paracoccus
		Paracoccus
E005-2-B	4	*Moraxella*
		Pseudomonas
		Bacillus
		Psychrobacter
E005-2-W3	1	*Cutibacterium*
E005-2-M8	5	*Rothia*
		Veillonella
		Neisseria
		Granulicatella
		Prevotella
E014-B	1	*Streptococcus*

MAGs were recovered from samples sequenced with 5GB of data using the Illumina platform.
